# Association between native T1 mapping of the kidney and renal fibrosis in patients with IgA nephropathy

**DOI:** 10.1186/s12882-019-1447-2

**Published:** 2019-07-11

**Authors:** M. P. Graham-Brown, A. Singh, J. Wormleighton, N. J. Brunskill, G. P. McCann, J. Barratt, J. O. Burton, G. Xu

**Affiliations:** 1 John Walls Renal Unit, University Hosptials of Leicester NHS Trust, Leicester, UK; 2 Department of Infection Immunity and Inflammation, School of Medicine and Biological Sciences, University of Leicester, Leicester, UK; 3 Deparment of Cardiovascular Sciences, University of Leicester and NIHR Leicester Cardiovascular Biomedical Research Centre, Glenfield Hospital , Leicester, UK

**Keywords:** IgA nephropathy, Renal fibrosis, Native T1 mapping, MRI

## Abstract

**Introduction:**

IgA nephropathy (IgAN) is the commonest global cause of glomerulonephritis. Extent of fibrosis, tubular atrophy and glomerulosclerosis predict renal function decline. Extent of renal fibrosis is assessed with renal biopsy which is invasive and prone to sampling error. We assessed the utility of non-contrast native T1 mapping of the kidney in patients with IgAN for assessment of renal fibrosis.

**Methods:**

Renal native T1 mapping was undertaken in 20 patients with IgAN and 10 healthy subjects. Ten IgAN patients had a second scan to assess test-retest reproducibility of the technique. Native T1 times were compared to markers of disease severity including degree of fibrosis, eGFR, rate of eGFR decline and proteinuria.

**Results:**

All patients tolerated the MRI scan and analysable quality T1 maps were acquired in at least one kidney in all subjects. Cortical T1 times were significantly longer in patients with IgAN than healthy subjects (1540 ms ± 110 ms versus 1446 ± 88 ms, *p* = 0.038). There was excellent test-retest reproducibility of the technique, with Coefficient-of-variability of axial and coronal T1 mapping analysis being 2.9 and 3.7% respectively. T1 correlated with eGFR and proteinuria (*r* = − 0.444, *p* = 0.016; *r* = 0.533, *p* = 0.003 respectively).

Patients with an eGFR decline > 2 ml/min/year had increased T1 times compared to those with a decline < 2 ml/min/year (1615 ± 135 ms versus 1516 ± 87 ms, *p* = 0.068), and T1 time was also higher in patients with a histological ‘T’-score of > 0, compared to those with a ‘T’-score of 0 (1575 ± 106 ms versus 1496 ± 105 ms, *p* = 0.131), though not to significance.

**Conclusions:**

Cortical native T1 time is significantly increased in patients with IgAN compared to healthy subjects and correlates with markers of renal disease. Reproducibility of renal T1 mapping is excellent. This study highlights the potential utility of native T1 mapping in IgAN and other progressive nephropathies, and larger prospective studies are warranted.

**Electronic supplementary material:**

The online version of this article (10.1186/s12882-019-1447-2) contains supplementary material, which is available to authorized users.

## Background

IgA nephropathy (IgAN) is the commonest global cause of glomerulonephritis. While mesangial IgA deposition is diffuse and global in IgAN, there is significant heterogeneity in the glomerular and tubulointerstitial response to this IgA deposition. While some patients have indolent, non-progressive disease, most have a steadily progressive decline in renal function despite current best medical therapies. It is estimated that between 30 to 40% of affected individuals will progress to end-stage renal disease over the course of 20 years [1, 2]. Common to other proteinuric renal diseases, decline in renal function is driven by the development and progression of interstitial fibrosis. The original Oxford Classification and all subsequent validation studies have consistently shown that interstitial fibrosis, tubular atrophy and glomerulosclerosis are the most reproducible predictors of progressive renal function decline in IgAN [3–5].

Renal biopsy is essential for a diagnosis of IgAN. In addition to providing a diagnosis, renal histomorphometry may be useful in prognostication and therapy selection [6, 7]. Unlike lupus nephritis and ANCA-associated vasculitis, where repeat renal biopsy is common to assess disease activity and response to immunomodulation, a repeat renal biopsy is not commonly performed in IgAN, principally due to the perceived poor risk benefit ratio [8, 9]. There have been only a few sequential renal biopsy studies to assess response to treatment in IgAN [10] (NCT02112838), however, a concern voiced by some investigators is the potential for sampling error, where biopsy samples are not necessarily representative of the whole kidney, adversely influencing any evaluation of treatment response [11]. Non-invasive screening tests that could assess the extent of interstitial fibrosis, or active inflammation are therefore highly desirable.

Native T1 mapping is a novel, non-contrast magnetic resonance imaging (MRI) technique which can characterize tissue with great specificity. Native T1 time is dependent on the molecular environment and total amount of water molecules within a given tissue and is influenced by a number of different factors. Myocardial oedema, protein deposition, and interstitial fibrosis all lengthen the myocardial T1 times, whilst iron overload and fatty deposits shorten T1 times. Myocardial native T1 has been shown to correlate well with histological fibrosis content in patients with aortic stenosis [12]. It is also able to identify and define myocardial inflammation in patients with myocarditis [13], aid in the diagnosis and in assessing response to treatment in patients with cardiac amyloidosis [14], and can differentiate abnormal disease states in a number of conditions [15, 16]. The possibility and potential uses of native T1 mapping to assess patients with chronic kidney disease (CKD) has been demonstrated [17] and T1 has been shown to be lengthened in patients with CKD compared to controls [18]. Studies have also suggested there may be a role for renal native T1 mapping in the assessment of transplant dysfunction [19], but no study has assessed renal native T1 mapping of subjects with IgAN.

The aims of this study were:

1. To compare renal cortical native T1 mapping in patients with biopsy proven IgAN to matched healthy subjects
2. To assess the test-retest reproducibility of renal native T1 mapping in patients with IgAN

To identify relationships between blood, urine and histological markers of renal disease and renal cortical native T1 mapping in patients with IgAN.

## Methods

### Study design and recruitment

This was a prospective, single-centre, observational pilot study conducted in the UK. The NHS Health Research Authority provided ethical approval and all participants gave written informed consent. All adult patients with biopsy proven IgAN with independent renal function and seen in within the last 12 months in outpatient clinics were given patient information sheets about the project, those who replied were contacted by research team on a first come first serve basis. Patients with renal transplantation or on renal replacement therapy were excluded. Twenty patients with biopsy-proven IgAN were recruited from general nephrology clinics at the Leicester General Hospital. In addition, 10 healthy subjects with no known kidney disease were recruited through local advertising within the hospital. Ten patients with IgAN underwent repeat MRI scan within 14 days of initial scan, to assess test-retest reproducibility of the technique. Patients were not fasted, and no instructions given regarding fluid intake prior to the scan.

### Investigations

All participants underwent the following investigations during a single visit: blood pressure, height and weight measurement, blood sampling for renal function, urine collection for protein creatinine ratio and an MRI scan as described below. Estimated glomerular filtration rate (e-GFR) was calculated using the Chronic Kidney Disease Epidemiology Collaboration (CKD-EPI) formula. Previous renal biopsy report was reviewed and a recognised IgAN morphological biopsy classification was applied Oxford-MEST classification [20, 21].

### Magnetic resonance imaging acquisition

MRI of both kidneys was performed on a 3-Tesla scanner (Magnetom Skyra, Siemens AG, Healthcare Sector, Erlangen, Germany), using an 18-channel phased-array body coil, and an identical protocol in patients and controls. Following localisers, T1 imaging was undertaken using a prototype, breath-held, end-expiratory, ECG-gated single-shot Modified Look-Locker Inversion Recovery (MOLLI) sequence [22] with the 3(3)3(3)5 sampling pattern, and the following typical parameters: slice thickness 5 mm, field of view 300 × 400 mm, flip angle 32°, minimum TI 120 ms, inversion-time increment 80 ms. MOLLI images of both kidneys were acquired in coronal plane, with the slice planned through the centre of the renal pelvis, after applying a tight shim. If artefacts were present, imaging was repeated after increasing the field of view or swapping the phase encoding direction. This was followed by acquisition of three axial slices covering the superior, mid and inferior poles of each kidney, ensuring the presence of cortex and medulla. The MOLLI sequence produces a series of 11 images with different inversion times, with data collected over 17 heart-beats. The Siemens software (Syngo MR D13) then employs inline reconstruction software to produce a T1 parametric map, with pixel-by-pixel computation of the T1 values [22].

For measurement of bipolar kidney length a T1 volume-interpolated breath hold examination (VIBE) sequence was used. Slices were positioned in the true axial plane to include both kidneys in a single breath-hold, with the following typical parameters: slice thickness 2.5 mm, no inter-slice gap, field of view 300 × 400 mm, flip angle 9°.

### Image analysis

**Fig. 1:**
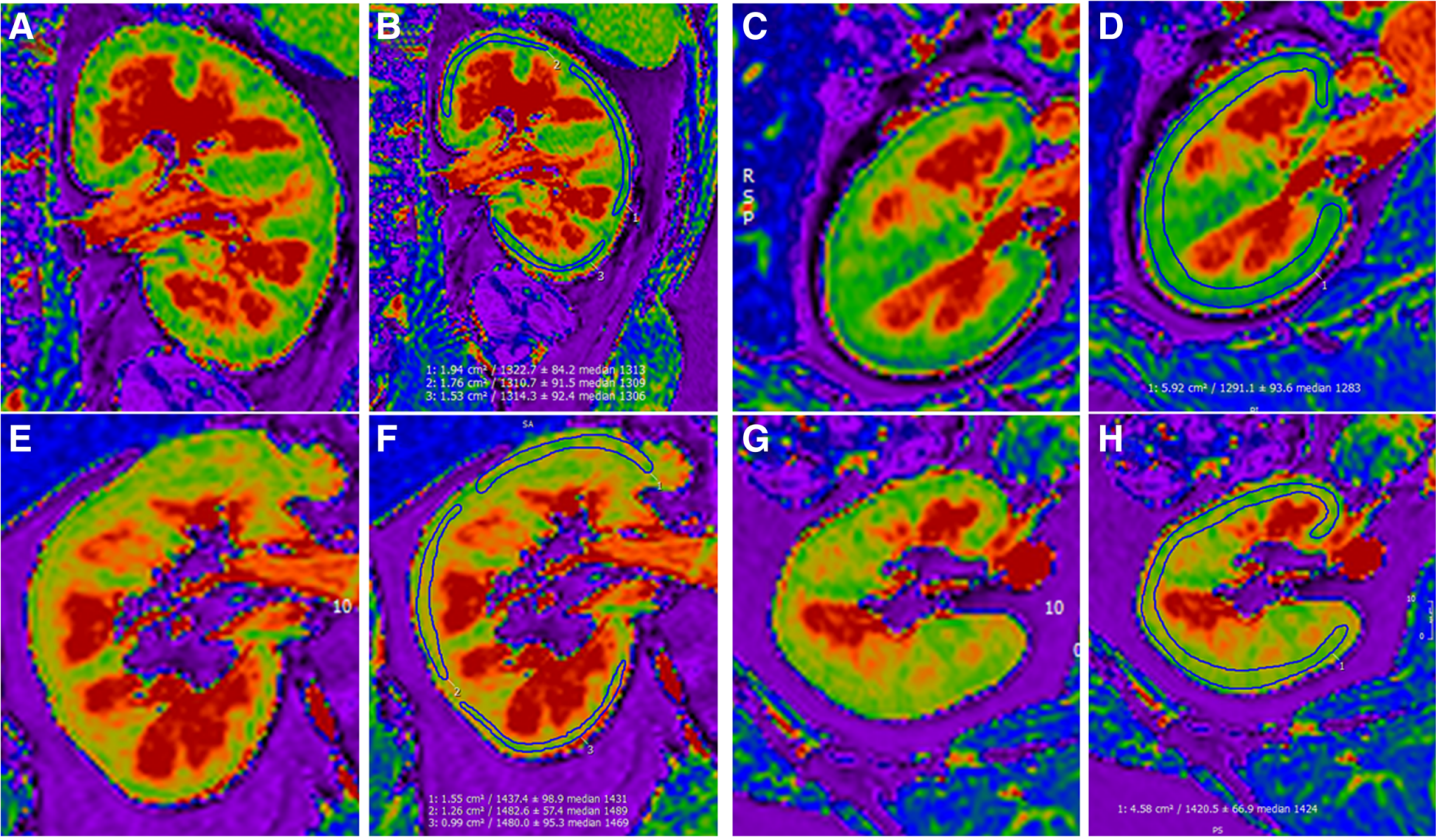
**a** Coronal native T1 map of the left kidney of a healthy subject. **b** Coronal native T1 map of the left kidney the same healthy subject with regions of interest contoured in renal cortex of the left upper pole, the left mid pole and the left lower pole to define regional cortical native T1. **c** Mid-kidney axial native T1 map of the left kidney of a healthy patient. **d** Mid-kidney axial native T1 map of the left kidney of the same healthy subject with region of interest contoured in renal cortex. **e** Coronal native T1 map of the right kidney of a patient with IgA nephropathy. **f** Coronal native T1 map of the right kidney of the same patient with IgA nephropathy with regions of interest contoured in renal cortex of the left upper pole, the left mid pole and the left lower pole to define regional cortical native T1. **g** Mid-kidney axial native T1 map of the right kidney of a patient with IgA nephropathy. **h** Mid-kidney axial oblique native T1 map of the right kidney of the same patient with IgA nephropathy with region of interest contoured in renal cortex

Renal native T1 parametric maps were used to calculate native T1, which was performed using CMR42 software (Circle Cardiovascular Imaging, Calgary, Alberta, Canada). On the coronal images, three regions of interest were manually drawn in the renal cortex, covering the superior, middle and inferior poles (Fig. 1). Regions of interest were also drawn in the axial slices to quantify the average cortical T1 time in the superior, middle and inferior poles of each kidney (Fig. 1). Using the T1 VIBE sequence a 3D reconstructed image of each kidney was acquired in the true bipolar long-axis. The true maximal bipolar kidney length could then be defined.

#### Statistical analysis

Data were analysed using IBM SPSS Statistics version 24.0 (IBM, Armonk, New York, U.S.A). Continuous variables are expressed as mean ± standard deviation. Normality of the data was checked using Shapiro-Wilk test. Patients with artefacts affecting regions of interest were excluded from analysis. Between group differences in T1 time and continues data was assessed using independent samples Student’s t tests, regional differences within an individual kidney was assessed using dependent T test, Chi-Squared was used to assess categorical data. Evaluation of correlation between MRI measurements and serum and urine biochemistry parameters was performed using Pearson’s correlation coefficient. Throughout, *p*-values < 0.05 were deemed significant. Reproducibility and agreement were evaluated with intra-class correlation coefficients (ICC) and Bland-Altman analysis.

## Results

Twenty patients with biopsy proven IgAN and 10 aged matched healthy subjects were recruited; the demographic data for each group is displayed in Table 1. All subjects tolerated the MRI scan and completed the full scan protocol. There was no significant difference in age, and blood pressure between groups, all participants were Caucasian. CKD-EPI eGFR as measured on the day of MRI scan was 62 ± 36 ml/min/1.72 m2 in the IgAN group and 91.5 ± 9.1 ml/min/1.72 m2 in the healthy subjects (*p* < 0.01). For patients with IgAN, mean time from most recent biopsy to MRI scan was 2.21 ± 1.65 years. All but 3 patients with IgAN were on either on ACEI/ARB at maximum tolerated dose and no patients were on immunosuppressive therapy. Neither patients nor healthy subjects had diabetes mellitus at the time of MRI scan. Mean rate of eGFR change in the patient group was − 3 ± 5.56 ml/min/year based on eGFR calculations for 2 or more blood tests 24 months prior to MRI scan.

**Table 1:** Baseline parameters. Baseline clinical and biochemical parameters are shown of healthy subjects and patients with IgA nephropathy. Results are shown as mean ± standard deviation

Parameter	Healthy volunteers (*n* = 10)	IgAN group (*n* = 20)	*p*-value
Age (years)	46 ± 16	44 ± 16	0.72
Gender (% Male)	60%	70%	0.58
Body mass index (kg/m^2^)	24.7 ± 2.5	29.6 ± 6.6	0.04
Blood pressure (mmHg)	117/71 ± 9/12	126/77 ± 12/7	.06/.09
Mean number of Antihypertensive	0.2	1.81	0.00
Total number of on ACE/ARB ^c^	1	17	0.00
CKD-EPI eGFR (ml/min/1.73m^2^)	91.5 ± 9.1	62 ± 36	0.02
Serum creatinine (μmol/L)	77 ± 12.2	151.7 ± 110	0.04
Protein Creatinine Ratio (mg/mmol)	7.13 ± 4.0	158.1 ± 201.5	0.03
Haemoglobin (g/L) ^b^	140 ± 12	140 ± 16^b^	0.99
Haematocrit (L/L)^b^	0.419 ± 0.03	0.416 ± 0.04^b^	0.84
T MEST score (number)^a^			
0		8	
1		6	
2		5	
S MEST score (number)^a^			
0		4	
1		15	
CKD staging (number of patients)			
*1*		5	
*2*		3	
*3*		9	
*4*		2	
*5*		1	

^a^19 Patients had MEST score for biopsy

^b^1 IgAN patient did not have full blood account carried out on the day of MRI

^c^ 1 patient in control group on Angiotensin Converting Enzyme/Angiotensin Renin blocker

### Renal cortical T1 times

**Fig. 2:**
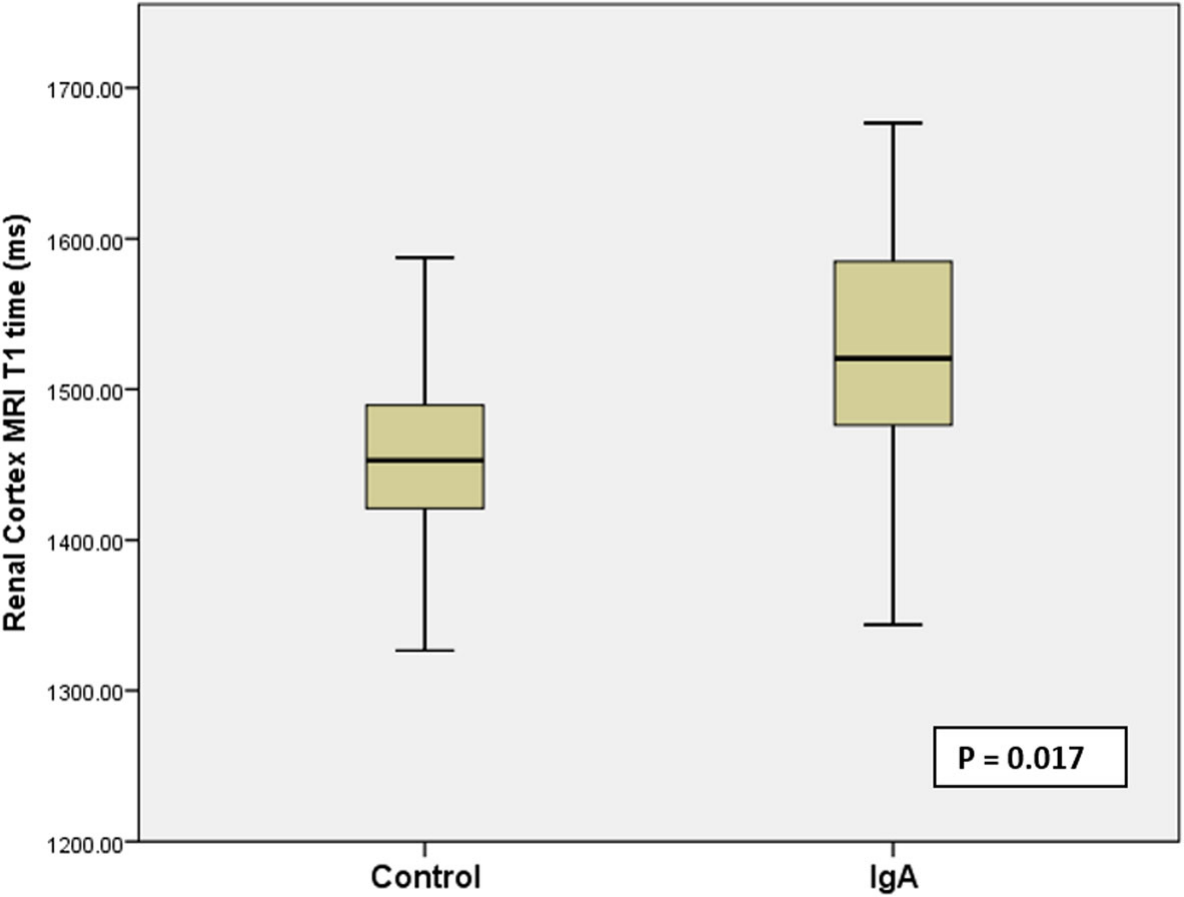
Mean native T1 from coronal images in patients with IgA nephropathy vs control subjects

Nineteen patients had analysable coronal T1 maps for both kidneys. Eighteen patients had analysable axial T1 maps for both kidneys. Mean cortical native T1 time for both coronal and axial images for patients with IgAN were significantly longer than for control subjects (1542 ± 107 ms versus 1443 ± 80 ms, *p* = 0.017 and 1540 ms ± 110 ms versus 1446 ± 88 ms for controls, *p* = 0.038 respectively) (Fig. 2). There were no differences in mean cortical T1 time between coronal and axial images for patients with IgAN (1541 ± 113 ms versus 1540 ± 110 ms, *p* = 0.906), or for control subjects (1438 ± 83 ms versus 1446 ± 88 ms, *p* = 0.531).

### Test-retest reproducibility

**Fig. 3:**
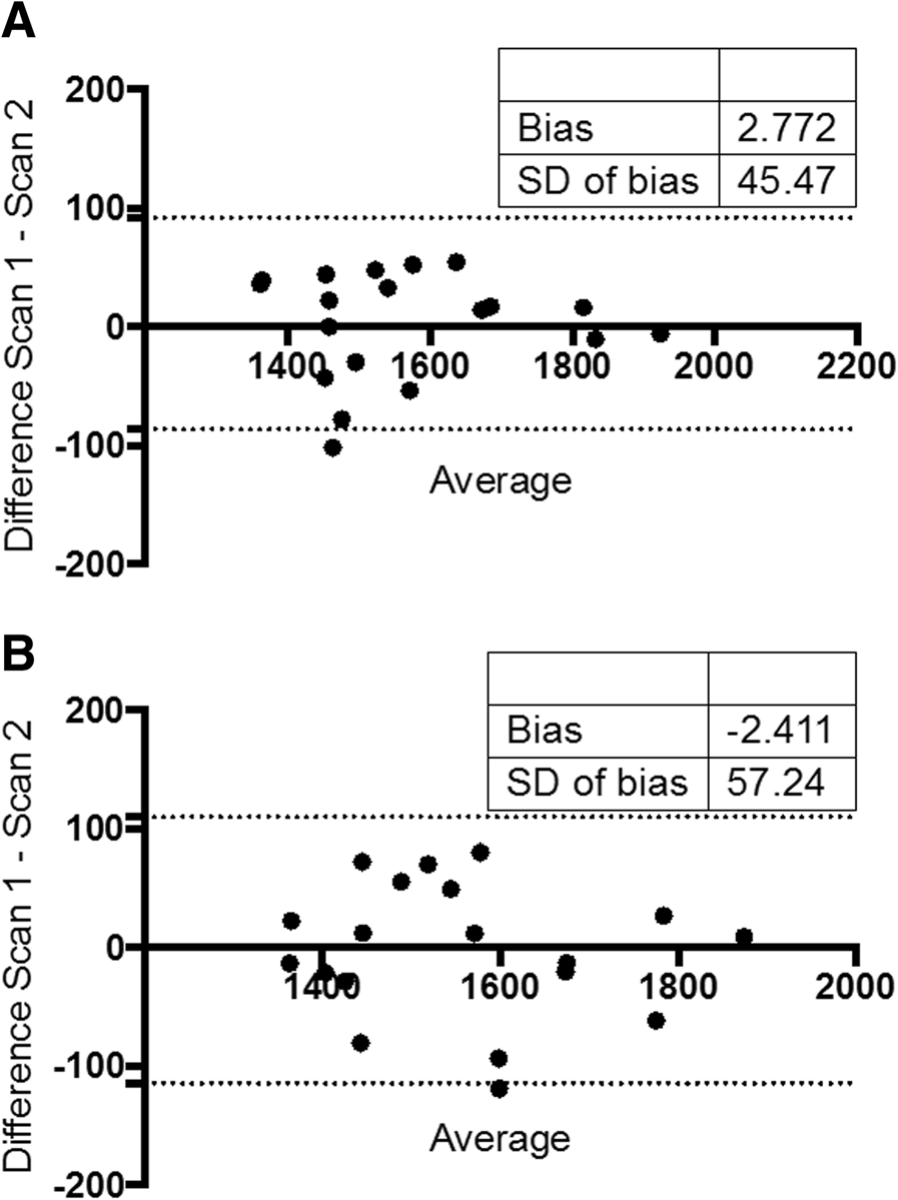
Bland-Altmann plots of test-retest reproducibility of native T1 mapping in patients with IgA nephropathy. **a** Coronal images, **b** Axial images

The mean time between the two MRI scans for patients undertaking the test-retest reproducibility study was 5.3 ± 5.8 days. One patient had multiple cysts on one kidney and was excluded from the analysis. Bland-Altman analysis of repeated sagittal and coronal native T1 maps did not reveal any evidence of systematic bias with all data plots falling within 95% confidence intervals (Fig. 3). There were strong correlations between test-retest renal native T1 times for sagittal and coronal images (*r* = 0.96 and *r* = 0.93 respectively) with excellent test-retest reproducibility (CoV of 2.9 and 3.68% respectively).

### Associations between clinical, biochemical and histological parameters with MRI

There were significant correlations between cortical native T1 time and eGFR, as well as proteinuria (*r* = − 0.444, *p* = 0.016 and *r* = 0.533, *p* = 0.003 respectively) (Fig. 3). Patients with IgAN who had an eGFR decline of > 2 ml/min/year over the preceding 24 months tended to have increased cortical coronal native T1 time compared to those with an eGFR decline of < 2 ml/min/year (1615 ± 135 ms versus 1516 ± 87 ms, *p* = 0.068). There were no significant correlations between age, haemoglobin or blood pressure and native T1 time. (Scatter plots to be added).

In IgAN patients, the mean cortical native T1 time was marginally reduced in patients with a histological ‘T’-score of 0, compared to patients with a histological ‘T’-score of > 0, though not to statistical significance (1496 ± 105 ms versus 1575 ± 106 ms, *p* = 0.131). There was no significant difference between native T1 time for patients with IgAN who had an ‘S’ score of 0 on histology, compared to those with an ‘S’-score of > 0 on histology (1507 ± 53 ms versus 1565 ± 136 ms, *p* = 0.402).

There was no difference in bipolar kidney length between patients with IgAN and healthy subjects (10.92 ± 0.86 cm versus 11.18 ± 0.37 cm, *p* = 0.36). There were no significant correlations between biploar kidney length and eGFR, proteinuria, rate of eGFR decline, or histological ‘T’-scores or ‘S’-scores. Additionally there was no correlation between native T1 time and bipolar kidney length in patients with IgAN.

## Discussion

For the first time we have shown that renal native T1 times are significantly higher in patients with biopsy-proven IgAN than age matched healthy subjects. We also demonstrate excellent tolerability and test-retest reproducibility of this novel technique. The absolute values for cortical native T1 time that we describe are very similar to those described in a previous study of patients with CKD conducted at 3-Tesla [18]. We have also shown similar relationships between cortical native T1 time in patients with IgAN and both eGFR and degree of proteinuria [18]. We have shown that MRI native T1 time is able to differentiate patients with progressive IgAN (eGFR decline > 2 ml/min/year) from those where decline in renal function is more stable. It is possible that native T1 mapping may be able to define the global degree of fibrosis in patients with IgAN, making it a potentially exciting non-invasive imaging biomarker for use in clinical studies.

Although we were unable to show a significant difference between native T1 times between patients with a histological T-score of ‘0’ compared to patients with a histological T-score of > 0, as this pilot study was not powered to do so, there was a trend, with an absolute difference of 79 ms between the groups. Larger studies will be needed to explore this further. Animal studies have shown that renal native T1 mapping correlates well with degree of fibrosis and inflammation on whole kidney histology [23], although it must be noted that the same study compared native T1 to degree of fibrosis in 4 human subjects and found the correlation to be significantly weaker, though still significant.

We have shown that the test-retest reproducibility of cortical native T1 mapping in patients with IgAN is excellent. The reproducibility of the coronal images was slightly superior to the axial images, with fewer areas of artefact requiring exclusion. Bipolar kidney length was not significantly different between healthy subjects and patients with IgAN and there were no relationships between bipolar kidney length and measures of renal function and renal disease (eGFR, proteinuria or histology). This further highlights the potential added benefits of tissue characterisation with native T1 mapping over-and-above macroscopic changes in kidney size and appearance.

We did observe some variability in native T1 times between different cortical regions especially in patients with IgAN there was more regional variability in the native T1 times, however the significance of this is unclear and need future investigation in a larger cohort (Additional file 1: Table S1).

In contrast to previous studies that have conducted renal T1 mapping in patients with renal disease [17–19] we recruited healthy subjects matched for age and gender. Blood pressure was well controlled in both groups and the groups were well matched for haemoglobin and haematocrit, both of which may influence native T1 times. The population of IgAN patients in this study were not on any immunomodulatory therapy, which may alter native T1 times via moderation of the inflammatory response, although there are currently no trial data to support this. Future, prospective studies should assess whether native T1 times change as the disease progresses, whether these changes associate with changes in histology and whether interventions have any effect on native T1 that relate to other markers of renal disease and outcomes.

### Limitations

The sample size of this pilot study is relatively small, although even with small numbers we have been able to demonstrate the potential utility of this technique. One of the major advantages of MRI scanning are its multi-parametric capabilities [17]. Previous studies that have looked at native T1 mapping in patients with renal disease have done so as part of a more comprehensive MRI protocol that has included measures such as arterial spin labelling, diffusion-weighted imaging, T2* imaging and renal artery and vein blood flow [17, 23]. We deliberately focussed on native T1 mapping in this study due to local expertise in the acquisition and the analysis of native T1 mapping [15, 22], but this does not mean future studies should not explore the roles of other MRI-derived measures. We deliberately focussed on patients with a single, defined renal disease, proven histologically to minimise confounding factors that may influence native T1 times. Fibrosis is the common end-point of many parenchymal renal diseases. Whilst the findings of this study are not automatically generalizable to all renal diseases, the potential for native T1 to characterise renal cortical fibrosis in related progressive nephropathies is clear and should and tested in other, defined, disease populations across the spectrum of CKD, preferably with histology. Another limitation of this study is the time between renal biopsy and the time of scan, but this study was not powered or designed to assess this, and findings are only exploratory in nature. Future studies should seek to conduct renal biopsy and MRI scan at the same time points.

## Conclusions

Renal cortical native T1 time is significantly increased in patients with IgAN compared to matched healthy subjects and correlates with markers of renal disease. Its is a well-tolerated technique with excellent test-retest reproducibility, and shows great potential as a non-invasive measure of interstitial fibrosis and inflammation in patients with IgAN and other renal diseases.

## Additional file

Additional file 1: **Table S1.** Regional differences in cortical native T1 times from axial and coronal native T1 maps within individual kidneys. * *P* < 0.05. (DOCX 20 kb)

## Data Availability

Technical appendix, statistical code, and dataset of the study is available upon request from the corresponding author.

## References

[1] Alamartine E, Sabatier JC, Guerin C, Berliet JM, Berthoux F (1991). Prognostic factors in mesangial IgA glomerulonephritis: an extensive study with univariate and multivariate analyses. Am J Kidney Dis.

[2] Koyama A, Igarashi M, Kobayashi M (1997). Natural history and risk factors for immunoglobulin a nephropathy in Japan. Research Group on Progressive Renal Diseases. Am J Kidney Dis.

[3] Daniel L, Saingra Y, Giorgi R, Bouvier C, Pellissier JF, Berland Y (2000). Tubular lesions determine prognosis of IgA nephropathy. Am J Kidney Dis.

[4] Mera J, Uchida S, Nagase M (2000). Clinicopathologic study on prognostic markers in IgA nephropathy. Nephron..

[5] To KF, Choi PC, Szeto CC, Li PK, Tang NL, Leung CB (2000). Outcome of IgA nephropathy in adults graded by chronic histological lesions. Am J Kidney Dis.

[6] Tesar V, Troyanov S, Bellur S, Verhave JC, Cook HT, Feehally J (2015). Corticosteroids in IgA nephropathy: a retrospective analysis from the VALIGA study. J Am Soc Nephrol.

[7] Trimarchi H, Barratt J, Cattran DC, Cook HT, Coppo R, Haas M (2017). Oxford classification of IgA nephropathy 2016: an update from the IgA nephropathy classification working group. Kidney Int.

[8] Preda A, Van Dijk LC, Van Oostaijen JA, Pattynama PM (2003). Complication rate and diagnostic yield of 515 consecutive ultrasound-guided biopsies of renal allografts and native kidneys using a 14-gauge Biopty gun. Eur Radiol.

[9] Stratta P, Canavese C, Marengo M, Mesiano P, Besso L, Quaglia M (2007). Risk management of renal biopsy: 1387 cases over 30 years in a single Centre. Eur J Clin Investig.

[10] Hou JH, Le WB, Chen N, Wang WM, Liu ZS, Liu D (2017). Mycophenolate Mofetil combined with prednisone versus full-dose prednisone in IgA nephropathy with active proliferative lesions: a randomized controlled trial. Am J Kidney Dis.

[11] Corwin HL, Schwartz MM, Lewis EJ (1988). The importance of sample size in the interpretation of the renal biopsy. Am J Nephrol.

[12] Bull S, White SK, Piechnik SK, Flett AS, Ferreira VM, Loudon M (2013). Human non-contrast T1 values and correlation with histology in diffuse fibrosis. Heart..

[13] Ferreira VM, Piechnik SK, Dall'Armellina E, Karamitsos TD, Francis JM, Ntusi N (2013). T(1) mapping for the diagnosis of acute myocarditis using CMR: comparison to T2-weighted and late gadolinium enhanced imaging. JACC Cardiovasc Imaging.

[14] Karamitsos TD, Piechnik SK, Banypersad SM, Fontana M, Ntusi NB, Ferreira VM (2013). Noncontrast T1 mapping for the diagnosis of cardiac amyloidosis. JACC Cardiovasc Imaging.

[15] Graham-Brown MP, March DS, Churchward DR, Stensel DJ, Singh A, Arnold R (2016). Novel cardiac nuclear magnetic resonance method for noninvasive assessment of myocardial fibrosis in hemodialysis patients. Kidney Int.

[16] Dass S, Suttie JJ, Piechnik SK, Ferreira VM, Holloway CJ, Banerjee R (2012). Myocardial tissue characterization using magnetic resonance noncontrast t1 mapping in hypertrophic and dilated cardiomyopathy. Circ Cardiovasc Imaging.

[17] Cox EF, Buchanan CE, Bradley CR, Prestwich B, Mahmoud H, Taal M (2017). Multiparametric renal magnetic resonance imaging: validation, interventions, and alterations in chronic kidney disease. Front Physiol.

[18] Gillis KA, McComb C, Patel RK, Stevens KK, Schneider MP, Radjenovic A (2016). Non-contrast renal magnetic resonance imaging to assess perfusion and Corticomedullary differentiation in health and chronic kidney disease. Nephron..

[19] Peperhove M, Vo Chieu VD, Jang MS, Gutberlet M, Hartung D, Tewes S (2018). Assessment of acute kidney injury with T1 mapping MRI following solid organ transplantation. Eur Radiol.

[20] Cattran DC, Coppo R, Cook HT, Feehally J, Working Group of the International Ig ANN, the Renal Pathology S (2009). The Oxford classification of IgA nephropathy: rationale, clinicopathological correlations, and classification. Kidney Int.

[21] Coppo R, Troyanov S, Camilla R, Hogg RJ, Working Group of the International Ig ANN, the Renal Pathology S (2010). The Oxford IgA nephropathy clinicopathological classification is valid for children as well as adults. Kidney Int.

[22] Singh A, Horsfield MA, Bekele S, Khan JN, Greiser A, McCann GP (2015). Myocardial T1 and extracellular volume fraction measurement in asymptomatic patients with aortic stenosis: reproducibility and comparison with age-matched controls. Eur Heart J Cardiovasc Imaging.

[23] Friedli I, Crowe LA, Berchtold L, Moll S, Hadaya K, de Perrot T (2016). New magnetic resonance imaging index for renal fibrosis assessment: a comparison between diffusion-weighted imaging and T1 mapping with histological validation. Sci Rep.

