# Supplementary material for: Association between native T1 mapping of the kidney and renal fibrosis in patients with IgA nephropathy
Source: BMC Nephrol. 2019 Jul 11;20:256. doi: 10.1186/s12882-019-1447-2 (PMC6621982; doi:10.1186/s12882-019-1447-2)
Supplement: Supplementary file 1 — Table S1. Regional differences in cortical native T1 times from axial and coronal native T1 maps within individual kidneys. * P < 0.05. (DOCX 20 kb) [file 12882_2019_1447_MOESM1_ESM.docx]

Additional file 1: Table S1 Regional differences in cortical native T1 times from axial and coronal native T1 maps within individual kidneys. * P <0.05

|  | **Regional T1 times of renal cortex (ms)** | | **p-value** |
| --- | --- | --- | --- |
|  | Axial Right Upper | Axial Right Mid |  |
| Healthy subjects (n=10) | 1441±80 | 1433±85 | 0.50 |
| IgAN group (n=18) | 1538±118 | 1549±122 | 0.30 |
|  | **Axial Right Mid** | **Axial Right Lower** |  |
| Healthy subjects (n=10) | 1433±85 | 1443±85 | 0.06 |
| IgAN group (n=19)* | 1546±119 | 1524±103 | 0.04 |
|  | **Axial Right Upper** | **Axial Right Lower** |  |
| Healthy subjects (n=10) | 1441±80 | 1443±85 | 0.87 |
| IgAN group (n=18) | 1538±118 | 1532±101 | 0.6 |
|  | **Axial Left Upper** | **Axial Left Mid** |  |
| Healthy subjects (n=10) | 1463±90 | 1463±90 | 0.34 |
| IgAN group (n=18) | 1563±137 | 1539±131 | 0.08 |
|  | **Axial Left Mid** | **Axial Left Lower** |  |
| Healthy subjects (n=9) | 1453±91 | 1453±91 | 0.71 |
| IgAN group (n=19)* | 1538±128 | 1571±135 | 0.02 |
|  | **Axial Left Upper** | **Axial Left Lower** |  |
| Healthy subjects (n=9) | 1463±90 | 1451±101 | 0.36 |
| IgAN group (n=18) | 1563±137 | 1575±138 | 0.23 |
|  | **Coronal Right Upper** | **Coronal Right Mid** |  |
| Healthy subjects (n=10) | 1407±86 | 1437±68 | 0.07 |
| IgAN group (n=19)* | 1521±112 | 1558±117 | 0.00 |
|  | **Coronal Right Mid** | **Coronal Right Lower** |  |
| Healthy subjects (n=10) | 1437±68 | 1447±78 | 0.13 |
| IgAN group (n=19) | 1558±117 | 1555±113 | 0.78 |
|  | **Coronal Right Upper** | **Coronal Right Lower** |  |
| Healthy subjects (n=10)* | 1407±86 | 1447±78 | 0.04 |
| IgAN group (n=19)* | 1521±112 | 1555±113 | 0.03 |
|  | **Coronal Left Upper** | **Coronal Left Mid** |  |
| Healthy subjects (n=10) | 1463±86 | 1452±99 | 0.42 |
| IgAN group (n=20) | 1552±142 | 1555±140 | 0.81 |
|  | **Coronal Left Mid** | **Coronal Left Lower** |  |
| Healthy subjects (n=10) | 1452±99 | 1455±93 | 0.83 |
| IgAN group (n=20) | 1555±140 | 1570±137 | 0.24 |
|  | **Coronal Left Upper** | **Coronal Left Lower** |  |
| Healthy subjects (n=10) | 1463±86 | 1455±93 | 0.51 |
| IgAN group (n=20) | 1552±142 | 1570±137 | 0.10 |
